# The Evolutions of Microstructure, Texture and Hardness of A1050 Deformed by HPT at the Transition Area

**DOI:** 10.3390/ma16134686

**Published:** 2023-06-29

**Authors:** Hongjun Ni, Chenchen Ding, Haoyu Wang, Shuaishuai Lv, Xingxing Wang, Yu Liu

**Affiliations:** 1 School of Mechanical Engineering, Nantong University, Nantong 226019, China; ni.hj@ntu.edu.cn (H.N.); 2210310005@stmail.ntu.edu.cn (C.D.); 2110110147@stmail.ntu.edu.cn (H.W.); lvshuaishuai@ntu.edu.cn (S.L.); wangxx@ntu.edu.cn (X.W.); 2 School of Mechanical, Materials, Mechatronic and Biomedical Engineering, University of Wollongong, Wollongong, NSW 2522, Australia

**Keywords:** high-pressure torsion, microhardness, microstructural evolution, texture evolution, A1050

## Abstract

High-pressure torsion (HPT) is an effective severe plastic deformation method to produce ultrafine-grained (UFG) and nanocrystalline (NC) materials. In the past, most studies have focused on the evolutions in the microstructure, texture and mechanical properties of HPT-deformed materials at peripheral regions. The corresponding evolutions at a special area were observed in this study to reveal the potential plastic deformation mechanism for face-centred cubic (FCC) material with high stacking fault energy. A decreasing trend was found in grain size, and the final grain size was less than 1 μm. However, close observation revealed that the general trend could be divided into different sub-stages, in which grain elongation and grain fragmentation were dominant, respectively. Additionally, microhardness demonstrated a non-linear increase with the development of plastic deformation. Finally, the microhardness reached a high level of ~64 HV. At the early stages of HPT, the C component was transformed into a cube component, suggesting the material flows around the shear plane normal (SPN) axis at these stages. However, finally they will be replaced by ideal simple shear orientations.

## 1. Introduction

The Hall–Petch equation demonstrates the relationship between material mechanical properties and their grain size [1]. Specifically, metallic materials with smaller grains generally exhibit better mechanical performances, such as a higher tensile strength [2], a greater hardness [3] and a superior wear resistance [4]. In the past two decades, ultra-fine-grained (UFG) and nanocrystalline (NC) materials [5], consisting of extremely fine grains and exhibiting high-density defects, have attracted the interest of the scientific community due to their excellent properties. Traditionally, severe plastic deformation (SPD) technologies were employed to produce UFG and NC materials [6]. Equal channel angular pressing (ECAP), accumulative roll bonding (ARB), and high-pressure torsion (HPT) are the three most widely used SPD techniques due to their capacity for use in grain refinement [7,8,9]. Among them, HPT has its unique advantages over its two counterparts. For example, [7,9] HPT is able to provide higher plastic strain in one step at room or elevated temperature [10], especially for brittle materials. Unlike other SPD techniques, such as cold rolling, ECAP and ARB processes, the frictional force between the sample surfaces and HPT die surface plays a critical role in introducing the plastic shear strain during rotation [11,12]. As a result, some have believed that HPT is a more promising technology for producing UFG and NC materials [13].

It was well known that the mechanical properties of metallic materials, such as strength and hardness, as well as nanoindentation and nanoscratch resistance, are essentially related to their microstructure and texture [14,15,16]. In order to the reveal potential plastic deformation mechanism of materials deformed by HPT, it is necessary to investigate the evolution of their microstructure and texture. Indeed, a number of experiment characterizations and advanced finite element simulations have already been conducted. Gunderov et al. studied the microstructural evolution of HPT-processed amorphous alloys and found that the formation of nanocrystallization depends on the chemical composition of alloys and HPT processing regimes [17]. Additionally, the influence of defects and dislocations on the electro-resistivity of HPT-deformed nickel have been investigated by Korznikova et al. [18]. Deng et al. [19] investigated the microstructure and phase transformation in pure titanium with the addition of Fe after the HPT process and found a great influence of impure Fe content, compressive pressure and the number of HPT rotations. Additionally, Wei and co-authors [20] have conducted a systematic simulation to investigate the influence of HPT on the crystallographic orientation or texture evolutions in aluminum and nickel single crystals.

In the past decades, many efforts have been devoted to revealing the deformation behaviour of materials processed by HPT. These have resulting in a deeper understanding of the relationship between microstructure and strain. For example, Xu and his group investigated the microstructural evolution of the face-centred cubic (FCC) material in the early stages of HPT and demonstrated a gradual evolution in microstructure with increasing strain [21]. However, their study involved only a limited number of plastic strain points in HPT. In contrast, our recent study, which investigated more strain points in both low and high strain levels, revealed non-linear relationships between microstructure and strain, and between hardness and strain for the same materials [22]. A similar phenomenon has also been found in HPT-processed Ti-6Al-4V alloy when the rotation number of HPT process is less than 2 [23].

So far, a number of questions about microstructure evolution during HPT still remain unclear. Traditionally, it was believed that the deformation behaviour in the early stage of HPT differed from the ideal mode of simple shear which was widely considered to be the typical pattern for torsion, similar to the deformation features of an equal channel angular rotation [24] or ECAP with an outer corner curvature [25]. Hafok and Pippan utilized nickel single crystals, which can avoid the influences of adjacent grains and boundaries on deformation behaviour in the early stages, to explore the microstructural and textural evolution of HPT-deformed materials at a low strain level. Interestingly, they found that the material flows around the shear plane normal (SPN) at the central area where the equivalent strain is relatively low [26]. In a situation with ideal simple shear, in contrast, the material flow is around the transverse direction (TD). This finding implies that there is a transformation of flow behaviour, namely from SPN mode to TD mode, as the strain gradually increases. However, most previous studies focused on the evolution of microstructure and texture at peripheral regions where the deformation behaviour is in line with that of simple shear mode.

Aluminum, AA1050, has a typical FCC crystal structure and has been selected widely as a model material for the investigation of texture evolution. As such, it has been subjected to deformation by different SPD methods. Thus, in the present work, the A1050 alloy has also been employed to study the microstructural and textural evolution during HPT in a wide strain range by studying the areas at a radial distance of 2 mm from the disc axis, i.e., the SPN axis. The microstructural and textural evolution at such a transition region is inevitably affected by the material flow at both the sample centre and the periphery area. By doing so, the corresponding evolution of microstructure and texture on the SPN-RD plane at the special area could be revealed.

## 2. Experiment

**Figure 1:**
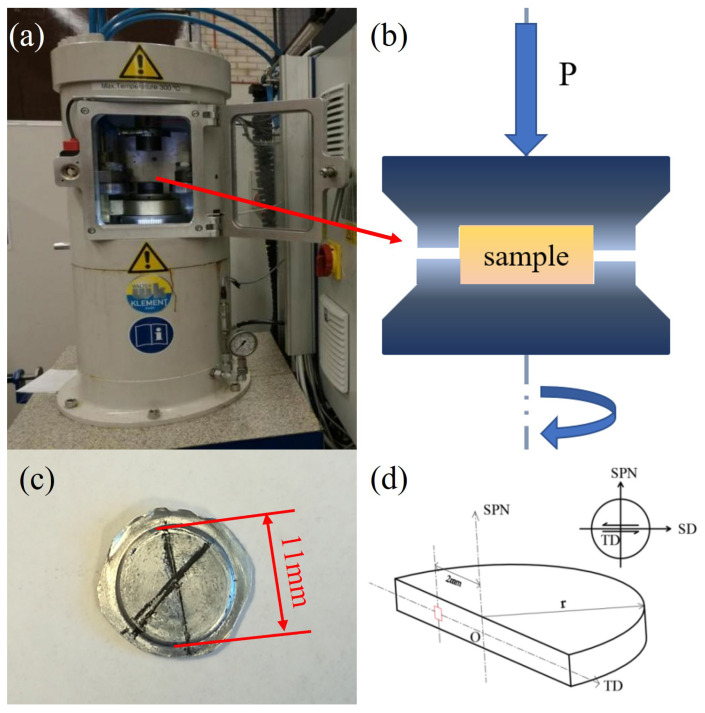
The HPT experiment of A1050. (**a**) The HPT equipment; (**b**) the schematic diagram of HPT experiment; (**c**) the HPT-deformed sample; (**d**) the schematic diagram of hardness testing and EBSD scanning area, and the sample reference system.

The composition of the commercial pure aluminium alloy employed in the research was: Fe: 0.31, Si: 0.06, Mn: 0.04, Mg: 0.01, Ti: 0.02, Zn: 0.04, Cu: 0.02, and Al: Bal (wt%). The HPT equipment (High pressure torsion press, Walter Klement GmbH, Bonn, Germany) is demonstrated in Figure 1a, and the schematic diagram in Figure 1b demonstrates the process of HPT. Specifically, the samples were placed between the upper and lower dies, then the lower die moved upward to generate a compressive pressure of ~6 GPa. Finally, the lower die was rotated to apply a torsional deformation on the samples. The dimension of the specimens that had been processed from an extruded rod was Φ10 × 1.35 mm after HPT. The heat treatment for these specimens was carried out under 300 °C for two hours to obtain recrystallized structures and then cooled in air. These samples were deformed by applying HPT under constrained conditions to 8 different torsional angles at ambient temperature and the corresponding equivalent strains calculated from equation ε = 2πnr/t√3 can be found in Table 1. A torsional speed of 0.5 rpm was used to minimize the heat effect caused by friction.

In order to conduct electron backscatter diffraction (EBSD) measurements, these samples were polished to be mirror-like surface by abrasive papers, polishing cloths and electrolytic polishing. The scanning electron microscope (JSM-7001F, Nippon Electronics Co., Tokyo, Japan), equipped with an Oxford EBSD detector, was used to measure crystallographic orientations. The accelerating voltage was 15 kV and the working distance was 15 mm. The EBSD scanning areas were indicated in Figure 1d and the scanning steps varied from 150–500 nm depending on the grain size. The grains comprising 10 or less pixels were removed during post-processing using Channel 5. Microhardness testing was undertaken at the same areas as EBSD measurements by using FM-810 (FUTURE TECH, Kanagawa, Japan) equipped with a Vickers indenter. At least three measurements at each testing point were carried out to obtain a reliable hardness value. The sample reference system is shown in Figure 1d. It should be noted that the SD, TD and SPN represent the shear direction, transverse direction and shear plane normal, respectively.

## 3. Result and Discussion

**Figure 2:**
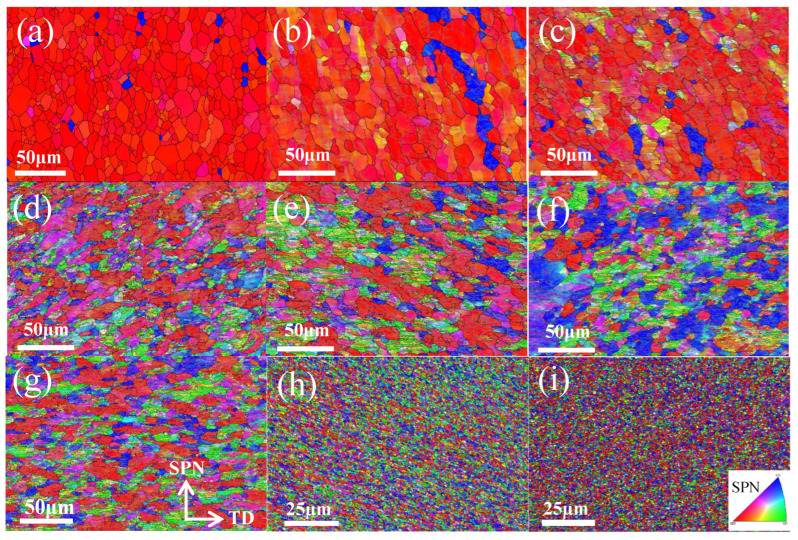
IPF-coloured EBSD maps of samples subjected to different torsional strains: (**a**) 0.00, (**b**) 0.21, (**c**) 0.42, (**d**) 1.27, (**e**) 2.54, (**f**) 5.08, (**g**) 10.16, (**h**) 15.24 and (**i**) 25.40.

**Figure 3:**
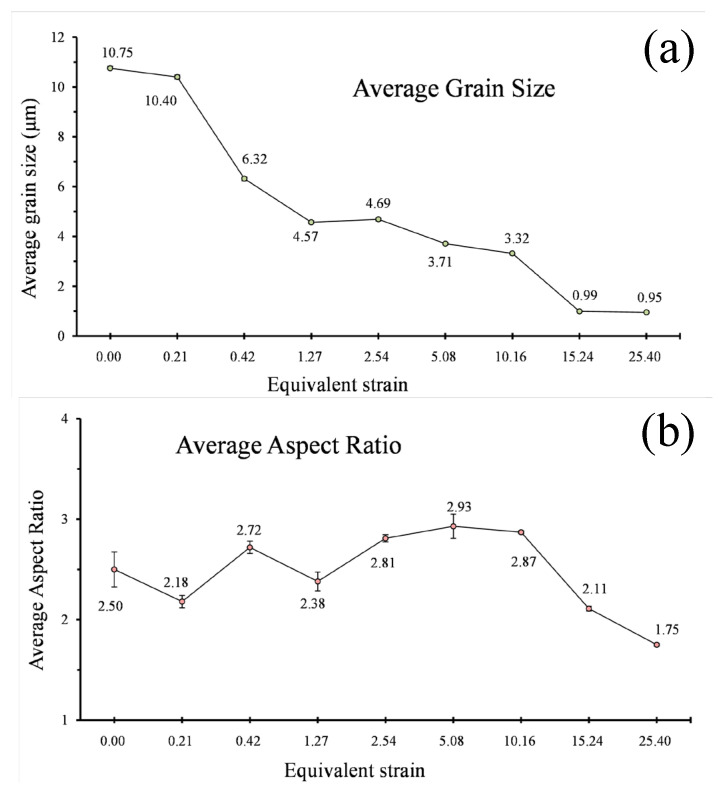
(**a**) The grain size and (**b**) aspect ratio of samples subjected to different torsional strains.

All EBSD maps of testing areas marked by red rectangles in the schematic diagram are demonstrated in Figure 2. These EBSD maps were measured on the SPN-TD plane and coloured according to the misorientation between crystallographic orientation and the SPN, thus it can be known that the red colour represents the {100} fibre according to the inset in Figure 2i. It can be found that the {100} fibre and annealing grain structures prevail in the unprocessed sample (Figure 2a) due to the effects of the extrusion process and heat treatment. The detailed grain information is demonstrated in Figure 3. Particularly, the grain size refers to the equivalent circle diameters of the detected grain and the grain aspect ratio is defined as the ratio between the longest axis and the shortest axis of a fitted ellipse in this study. It seems that the initial grain size and grain aspect ratio of the annealing samples are 10.75 μm and 2.50, respectively.

**Figure 4:**
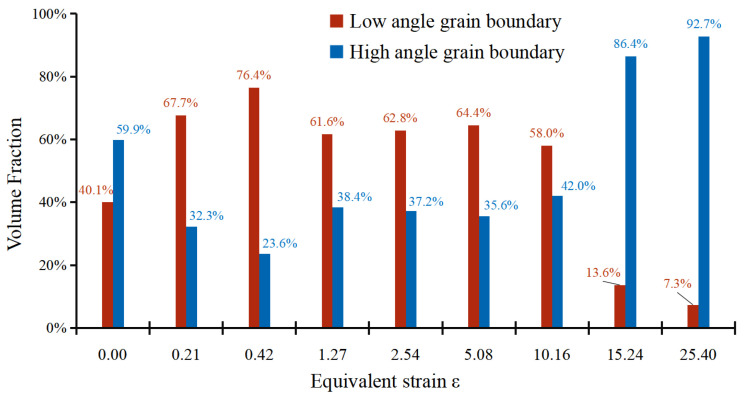
The fraction of low-angle grain boundary and high-angle grain boundary.

**Figure 5:**
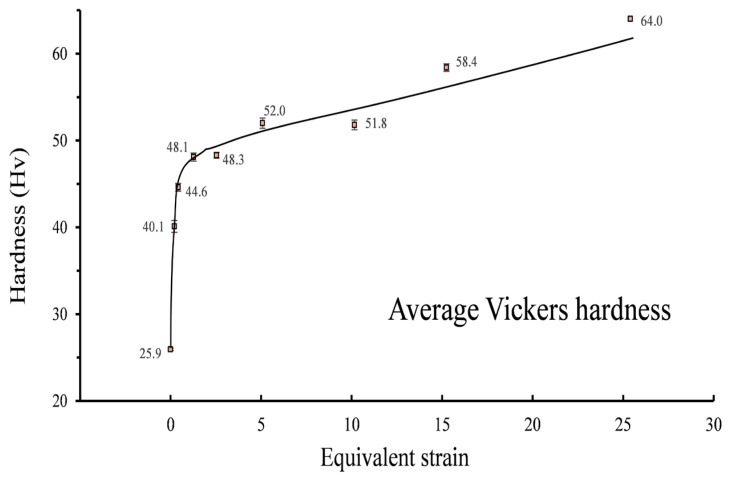
Microhardness of testing areas in the samples deformed by different equivalent strains.

As the equivalent strain increases slightly to ε = 0.21, the corresponding grain size drops somewhat to around 10.40 μm, and close inspection of EBSD maps reveals the potential mechanisms behind this. As shown in Figure 2b, most of the grains largely retain their original shape and colour in spite of the emergence of new colours and discontinuous substructures within many grains, leading to only a slight drop in grain size at this stage. The change in grain aspect ratio should be attributed to the applied high compressive pressure, which caused the reduction in sample thickness from 1.5 to 1.35 mm. As a result, the average aspect ratio declines to around 2.18. The enlargement of colour gradation indicates the development of substructures. Compared with the EBSD map of annealing specimens, a great number of sub-grain boundaries can be observed at the second stage, resulting in a dramatic increase in the fraction of low-angle grain boundaries at the same stage (Figure 4). The phenomenon can also be corroborated by the sample microhardness profile. The microhardness of different samples is plotted as functions of equivalent strain in Figure 5. A general upward trend can be found with the increase in turns of torsion. The microhardness value of the undeformed sample was 25.9 Hv, and then the microhardness value surged to about 40.1 Hv after a small amount of torsional strain (ε = 0.21). However, the same increment in equivalent strain (from 0.21 to 0.42) at the second stage only made the microhardness value increase by 4.5 HV. Therefore, it is reasonable to assume that the dramatic increase at first stage should be attributed to the high compressive pressure rather than to torsional deformation. Generally, the hardening mechanisms mainly link with grain boundaries and dislocation for the same group of samples [27,28]. According to Su et al. [5], vacancy-type defects induced by SPD also affect the mechanical properties of nanostructured metals. Therefore, the similar grain size at the two stages implies that the rapid dislocation multiplication and the formation of sub-structure owing to the large compressive force may be responsible for the sharp increase in microhardness. Such an inference can be verified by the continuous increase in low-angle grain boundaries at the early stage of HPT deformation (Figure 4).

**Figure 6:**
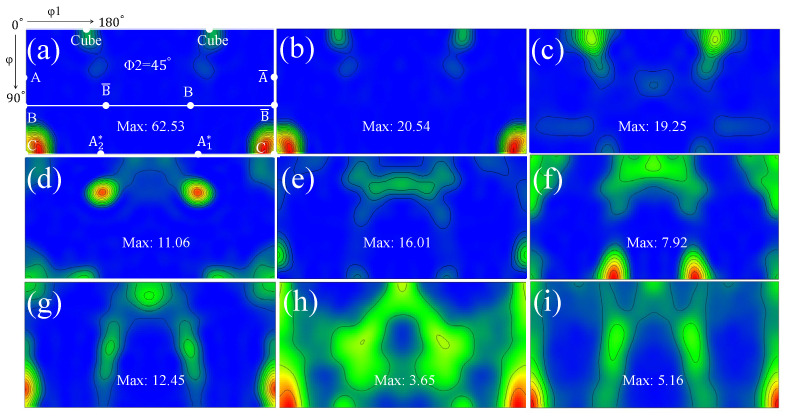
The ϕ2 = 45° ODF maps of samples subjected to different torsional strains: (**a**) 0.00, (**b**) 0.21, (**c**) 0.42, (**d**) 1.27, (**e**) 2.54, (**f**) 5.08, (**g**) 10.16, (**h**) 15.24 and (**i**) 25.40.

In addition to the stable grain size and the increased microhardness, the development of substructures gives rise to the transformation in texture as well. The ϕ2 = 45° orientation distribution function (ODF) maps of samples are illustrated in Figure 6. It is well known that the ideal simple shear texture components in f.c.c materials comprise A/A_b_, B/B_b_, $A_{1}^{*}$, $A_{2}^{*}$ and C [29,30]. It can be found that the initial specimen is predominated by C and cube components. The emergence of new colours and substructures which denote the grain rotation and the slip system activation indeed diminishes the strength of original texture, despite original components still being the predominant orientations. Specifically, the newly born grains generated from the requirements of strain compatibility and possessing different crystal orientations from original ones cause the maximum orientation density to drop from 62.53 to 20.54 at the first stage. Similar texture evolution has also been observed in the ECAP of pure aluminum alloy [29], which also reveals the significance of cryogenic deformation temperature in suppressing shear texture development. The formation of different shear texture components is attributed to the variation in slip system activities [11,24,25]. A higher degree of crystal rotation is generally observed at a higher plastic strain.

A dramatically unilateral decline in grain size is observed, with a further increase in the equivalent strain to ε = 0.42 and 1.27. At the second stage (ε from 0.21 to 0.42), many fine grains are formed, especially in the vicinity of grain boundaries, and several parent grains fragment into many segments. These factors should cause reductions in both grain size and in aspect ratio. However, grains are actually elongated towards the direction of shear force at the same time. The influence of grain elongation outweighs that of grain fragmentation. As a result, the aspect ratio rises from 2.18 to 2.72 at the second stage. Then, at the third stage, the original annealing structures cannot be distinguished from now onwards. The significant grain fragmentation leads to the grain size dipping rapidly to 4.57 μm. Simultaneously, as shown in Figure 6d, the divergence of grain orientation caused by grain fragmentation diminishes the initial orientation and weakens the orientation density. A significant decrease in the fraction of low-angle grain boundaries can been found at this stage because many sub-structures evolve into high-angle grain boundaries by absorbing dislocations, causing the corresponding drop in grain size [3,19,23].

A stable stage of grain size and microhardness can be detected when the equivalent strain ε increases from 1.27 to 2.54. This result is in good agreement with the Hall–Petch equation and indicates that the grains do not undergo substantial fragmentation. Thus, the corresponding orientation densities remain stable. Care must be taken to observe the slight grain growth at this stage, which is due in large part to shear-induced grain boundary motion rather than discontinuous dynamic recrystallization [31]. In the meantime, the aspect ratio, which jumps up ~18% to 2.81, shows a different trend from grain size and microhardness owing to the occurrence of continuous elongation during HPT without significant fragmentation.

A new round of grain refinement is initiated when the equivalent strain ε grows up from 2.54 to about 5.08 and 10.16. During the period, the grain size descends from 4.69 to 3.71 and 3.32 μm, respectively. At the former stage, the decline in grain size is accompanied by a corresponding increase in microhardness, i.e., from 48.3 Hv to 52.0 Hv. Interestingly, it is found that the growth trend in microhardness does not continue with the reduction in grain size at the latter stage. Stable microhardness and the slight decrease in grain size indicate the decrease in sub-structural density. During this stage, the fraction of low-angle grain boundaries drops marginally. As a typical FCC material, essentially, the rapid dislocation multiplication, movement and entanglement in A1050 with straining cause the formation of dislocation walls and cells [32,33]. Such dislocation walls and cells gradually evolve into low-angle grain boundaries and then into high-angle grain boundaries by absorbing dislocations of the same sign. During the process, the density of intragranular dislocation decreases to a low level because of the absorption. As a result, the value of microhardness is stabilized at about 52.0 Hv, although the grain size approximately decreases by 12%. Besides, the synergy of grain fragmentation and elongation leads to a fluctuation in grain aspect ratio which levels out at about 2.90. In terms of texture, several ideal orientations of simple shear, such as $A_{1}^{*}$ and $A_{2}^{*}$ components, begin to appear at the strain level. The $A_{1}^{*}$ and $A_{2}^{*}$ components transform into C component as the equivalent strain increases from 5.08 to 10.16, and the maximum orientation density becomes strong again.

As the equivalent strain increases to 15.24, the grain size plummets into 0.99 μm, representing the severe fragmentation of grains. The average grain size is less than 1 μm and falls into the region of UFGs from this strain level onwards. Extensive past research confirmed that HPT is able to produce UFG [34,35] and even NC materials [4,23,36,37]. In fact, the chemical composition plays a critical role in determining the final grain size in the samples deformed by HPT [13,19]. As a high stacking fault energy material, NC of A1050 is more difficult to generate via the SPD method compared to other alloys due to the rapid balance between grain fragmentation and grain growth [38,39]. Su et al. [40] studied the grain size of A1050 processed by ARB and ECAP and found that the ultimate sizes are 300 and 608 nm, respectively. In addition, the corresponding microhardness are 63.7 and 48.8. These data are of an order of magnitude to those obtained by HPT, indicating HPT is an effective SPD method. The substantial fragmentation of grains inevitably induces sharp decreases in grain aspect ratio to around 2.11 and surges in microhardness to 58.4 HV. The fractions of high-angle grain boundaries surge to high levels at the last two stages. The grain fragmentation alleviates the orientation density and leads to the transformation of texture. To be specific, the maximum value drops to about 3.65, while B/B_b_ and C components become the preferred orientations at this stage. At the final stage, a huge increment in equivalent strain only leads to a limited decline in grain size. However, the decline in aspect ratio and increase in microhardness indicate the developing substructures. Anyhow, the typical simple shear texture components can be seen in Figure 6i, implying the achievement of a dynamic equilibrium stage. It is worth mentioning that a more direct understanding of the grain refinement and shear texture evolution during the HPT process of AA1050 has been obtained from the current experimental study via a comparison with those of crystal plasticity finite-element simulations of HPT process [8,18].

## 4. Conclusions

A special area at a radial distance of 2 mm from the disc axis was chosen to reveal the evolutions of microstructure, texture and microhardness of A1015 subjected to HPT deformation at the transition area. As such, the following can be concluded.

(1) The results show that the HPT technique can reduce the grain size of commercial pure Al to less than 1 μm, which is of the same order of magnitude as the grain sizes obtained by ECAP and ARB, and produce UFG materials.
(2) The process of grain refinment is achieved by the repeated processes of grain elongation and subsequent grain fragmentation. Finally, the grain fragmentation and grain growth are balanced.
(3) At the low strain stage, microhardness value surges to a high level owing to the increase in substructure density, whereas the grain size hardly changes. Then, the microhardness levels out at around 48 Hv and the grain size reaches a plateau as the plastic strain increases. Finally, the microhardness rises steadily with the decline in grain size.
(4) When the equivalent strain increases from 0 to 0.42, it can be seen that the maximum orientation density of C component drops while that of Cube component increases. Such transition in texture components implies that the material flows around the SPN at these stages. However, such a flow pattern only has an effect at the early stage of HPT deformation. These initial components will evolve into typical ideal simple shear orientations with the development of HPT.

## Figures and Tables

**Table 1:** Equivalent strains of testing areas of the samples deformed by different angels.

Rotation angle θ (deg.)	15	30	90	180	360	720	1080	1800
Equivalent strain ε	0.21	0.42	1.27	2.54	5.08	10.16	15.24	25.40

## Data Availability

Not applicable.
